# Amino acid substitutions in the E2 glycoprotein of Sindbis-like virus XJ-160 confer the ability to undergo heparan sulfate-dependent infection of mouse embryonic fibroblasts

**DOI:** 10.1186/1743-422X-7-225

**Published:** 2010-09-14

**Authors:** Wuyang Zhu, Shihong Fu, Ying He, Jinping Li, Guodong Liang

**Affiliations:** 1 State Key Laboratory for Infectious Disease Prevention and Control (SKLID), Institute for Viral Disease Control and Prevention, China CDC, Beijing, China; 2 Department of Medical Biochemistry and Microbiology, University of Uppsala, The Biomedical Center, Uppsala, Sweden

## Abstract

We have recently demonstrated an essential role of the domain of 145-150 amino acid in the E2 glycoprotein of Sindbis virus in the interaction with cellular heparan sulfate (HS) and in the infection of mouse embryonic fibroblasts (MEF) cells. In this study, we constructed and characterized the mutants of Sindbis-like virus XJ-160 in which Tyr-146 and/or Asn-149 in the E2 glycoprotein had been substituted with His and Arg, respectively. Unlike parental virus XJ-160, mutants with either or both substitutions were able to infect wild-type mouse embryonic fibroblasts (MEF-*wt*) or MEF-*Epi^-/- ^*cells which produce mutant HS. Significantly more infectious particles were released from MEF-*wt *than from MEF-*Epi^-/- ^*cells. The mutant virus with both substitutions release was inhibited by pre-incubation of virus with heparin or pre-treatment of BHK-21 cells with HS-degrading enzyme. Both XJ-160 and the mutant viruses retained substantial neurovirulence in suckling mice. Our findings provide further support to the importance of positively charged residues in the HS-binding site of E2 in mediating Sindbis virus infection of MEF cells.

## Findings

Sindbis virus (SINV) is considered the prototype of *Alphavirus *genus, *Togaviridae *family [1,2]. Nearly 30 members of the genus are widely distributed in all continents except in the Antarctic. Sindbis virus is an enveloped virus with an 11.5 kb genome of single stranded RNA. The viral genome with a 5' terminal methylguanylate cap and a 3' terminal polyadenylate tail encodes four nonstructural proteins (nsP1-4) and three mature structural proteins (capsid, E2 and E1). Based on the divergence of nucleotide sequencing and biological characteristics, Sindbis virus can be divided into two groups, SINV and Sindbis-like virus (SINLV) [3]. SINV YN87448 and SINLV XJ-160 were isolated from a pool of Anopheles mosquitoes collected in Xinjiang and from a female patient with fever in Yunnan, China [4,5].

Heparan sulfate (HS) is a complex polysaccharide expressed in the form of proteoglycans on the surfaces of a wide range of invertebrate and vertebrate cells. Recently, HS has been found to be involved in the infection and pathogenicity of SINV [6,7] and other alphaviruses, such as Venezuelan encephalitis virus (VEEV), Semliki Forest virus (SFV) and Ross River virus (RRV) [8,9]. These investigations indicate that HS-dependent infection is an adaptation through the mutation for positively charged amino acid (aa), which frequently arise in laboratory strains during repeated passaging culture, and that wild-type strains of SINV might not bind well to HS. Besides alphaviruses, HS has been shown to serve as a receptor of a number of viruses, including herpes simplex virus (HSV) [10], human immunodeficiency virus type 1 (HIV-1) [11], adeno-associated virus type 2 (AAV2) [12], respiratory syncytial virus (RSV) [13], foot-and-mouth disease virus (FMDV) [14], and human papillomavirus type 11 [15].

Based on the difference in HS-dependent infectivity between YN87448 virus and XJ-160 virus, we have confirmed that interaction of E2 protein with HS is crucial for cellular infection of SINV [16]. Importantly, specific interaction of E2 peptide from YN87448 with heparin further suggests that the domain of 145-150 amino acid (aa) from the E2 gene may be a molecular basis for the specific interaction of SINV with cellular HS. Alignment of the E2 glycoprotein sequences from YN87448 and XJ-160 revealed the differences at the domain where the two positively charged aa (His and Arg at 146 and 149, respectively) of SINV YN87448 are neutral aa in SINLV XJ-160. This may explain that SINLV XJ-160 is not HS-dependent in infection of cells due to lacking of the two basic amino acids in the second HS-binding domain. Specific interaction of the peptide containing 145-150 aa from YN87448 E2 gene with heparin and no binding of the corresponding peptide from the of XJ-160 E2 gene to heparin further strengthened this speculation. However, the effects of E2-146Tyr and E2-149Asn on HS binding of Sindbis virus in the context of virus-RNA remain to be confirmed.

To find out the effect E2-146Tyr and E2-149Asn on HS-dependent infecion, three mutant viruses, BR-146 H containing point mutation 146Y-H, BR-149R containing 149 N-R and BR-HR containing both mutations were generated by *in vitro *transcription and electroporation method as previously described [16,17]. The results of immunofluorescence assay (IFA) and plaque assay indicated that XJ-160 virus was capable of assembling infectious particles in spite of different site-directed mutation at residue E2-146 or residue E2-149, and that all the mutant viruses displayed plaque morphologies similar to those formed by XJ-160, although the mutants seem to form bigger plaques (Additional file 1, Figure 1). In addition, determination of the titers demonstrated that BR-HR virus released more infectious particles than either parental virus XJ-160 or other two mutants (Additional file 2, Figure 2).

**Figure 1:**
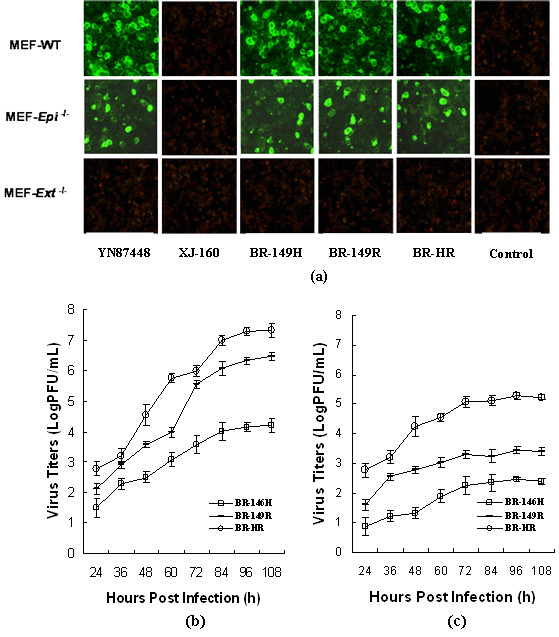
**Infectivity of recombinant viruses on MEF cell**. (a) Comparison of viral infections to MEF cells. MEF cells were grown on cover slips to 80% confluent and infected with YN87448 and XJ-160 as well as the mutants for 48 hours. Antiserum against YN87448 (used in YN87448) or XJ-160 (used in XJ-160 panel and each of recombinant virus) diluted1:100 were applied for IFA as previously described [16]. Non-infected MEF cells were used as control; Monolayer of MEF-wt (b) and MEF-Epi-/-cells (c) were infected with recombinant or parental viruses at a MOI of 0.01, the medium (1 ml) was removed at the indicated time points and evaluated for virus titer by plaque assay. Each point represents the mean ± SD of three wells.

**Figure 2:**
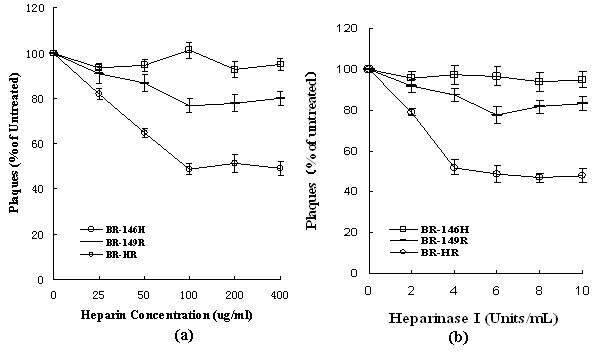
**Effect of heparin or heparinase I treatment on plaque formation of mutant viruses**. (a) Viruses diluted to 100-200 PFU/200 μl were incubated with heparin at the concentrations indicated for 1 h at 37°C. Then the plaque assay was performed on BHK-21 cells as previously described [16]. (b) Confluent BHK-21 cell monolayers were treated with heparinase I at the concentrations as indicated. After washing three times with PBS, the cells were infected with viruses diluted to 100-200 PFU in 200 μl. Plaque formation was analyzed as described.

To investigate the correlation between the mutants and cellular HS, we have compared infectivity of the mutants and XJ-160 virus in wild-type mouse embryonic fibroblast (MEF-*wt*) cells with that in two mutant MEF cell lines. The MEF-*Ext^-/- ^*is derived from the mice that are deficient in one of the HS polymerases, EXT1 [18], and the MEF-*Epi^-/- ^*is generated from mice that are lacking one of the modifications enzymes, glucuronyl C5-epimerase [19]. Characterization of the MEF cells generated from the mutant embryos illustrated that the MEF-*Ext^-/- ^*expressed a HS that is normal in composition, but is significantly shorter in chain length (20 kDa), in comparison to the wild-type HS (70 kDa) [20]; while the MEF-*Epi^-/- ^*cells expressed a full-length HS with a defective structure, e.g. lacking iduronic acid units and 2-O-sulfation accompanied with increased N-sulfation [21]. And three MEF cell lines (MEF-*wt*, MEF-*Epi^-/ ^*and MEF-*Ext^-/- -^*) used in this study was kindly provided by Dr Jin-ping Li, University of Uppsala, Sweden. MEF-*Ext^-/- ^*cells were resistant to all of viruses, likely due to the shorter HS chains expressed on their cell surface, indicating that the chain length of HS on cell surface is critical for viral attachment. In contrast, YN87448 and all the mutants were able to infect MEF-*wt *and MEF-*Epi*^-/- ^cells as demonstrated by IFA ( Figure 1a). These results suggest that substitution at residue E2-146 or residue E2-149 could help XJ-160 virus to overcome the MEF infection block by discriminating cellular HS.

It should be point out that either the mutants or YN87448 displayed considerably weaker infectivity in MEF-*Epi*^-/- ^cells in comparison to that in MEF-*wt *cells (Figure 1a). Quantification of the mutant viruses in culture medium clearly revealed different kinetics of viral reproduction in MEF-*wt *cells and MEF-*Epi*^-/- ^cells. All the mutants reach growth plateau approximately 84 h post-infection (h p. i.), but the reproduction ability varies, in the order of BR-HR, BR-149R and BR-HR in either MEF-*wt *cells or MEF-*Epi*^-/- ^cells (Figure 1b,c). More importantly, each virus released 2-3 order of magnitude more infectious particles in MEF-*wt *cells than that in MEF- *Epi*^-/- ^cells (Figure 1b,c). In consideration of the fact that 2-*O*-sulfated iduronic acid residue is commonly found in the HS sequences that interact with proteins (16), the reduced infectivity of the viruses to MEF-*Epi*^-/- ^cells in comparison to the MET-*wt *cells is likely due to the structural alterations of the surface HS that has a weaker interaction with E2 protein, suggesting that the fine structure of HS on cell surface is critical for viral attachment and infection. Indeed, the mutant HS from MEF-*Epi*^-/- ^exhibited an aberrant interaction with growth factors. Further studies to test the infection of the viruses on a 2-*O*-sulfotransferase mutant cell may provide additional information to this end.

Heparin, an analog of HS, is commonly used as a replacement of HS in in vitro experiments [22] and has been reported to be able to block Sindbis virus to infect and form plaques in cells [7]. Bacterial produced heparinase I that cleaves heparin and cellular HS has been commonly used to remove cell surface HS for various biological studies [23]. To further identify the functions of substitution at E2-146 or E2-149 on HS-dependent infection, we applied heparin or heparinase I to pre-incubate virus or BHK-21 cell, respectively. Pre-incubation of heparin with the viruses resulted in different inhibition of viral infection in BHK-21 cells. BR-149R and BR-HR showed a about inhibition of 20% and 50% at the concentration of 100 μg/ml, while addition of heparin had marginal effect on the plaque formation of BR-146 H (Figure 2a). Together with the similar effect of heparinase I treatment (Figure 2b), we can conclude that the simultaneous substitutions at E2-146Tyr and E2-149Asn enhance HS-dependent infection by XJ-160, especially substitution of E2-149 N-R plays an important role in HS-dependent infection. In the absence of crystal structural information about the *Alphavirus *E2 glycoprotein, it is difficult to predict that any particular amino acid is part of a binding site for HS or even that the amino acid is exposed on the surface of the virion. However, together with the notion that binding sites of SINV to HS are composed of the linear HS-binding domains and the scattered positively charged aa [16], our data demonstrate the importance of positively charged residues in the HS-binding domain of E2 in mediating SINV infection.

**Figure 3:**
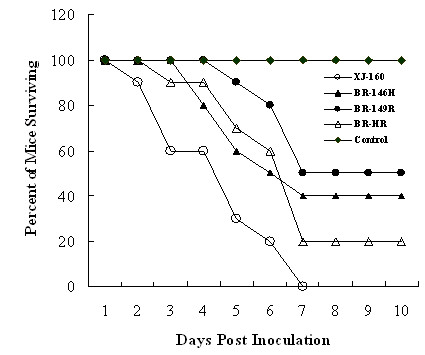
**Neurovirulence of the viruses for suckling mice**. Suckling mice (three days old) were inoculated intracerebrally with 30 μl of 10^3 ^PFU/ml recombinant or parental viruses; equal volume of Eagle's fortified with 1% fetal bovine serum was used as control.

Increased binding of alphaviruses to HS usually results in smaller plaque size under agar, more rapid clearance from the blood, and higher neurovirulence when inoculated directed into the brain of mice [16,24-27]. Here, we show that the mutants with partial HS-dependent phenotype in comparison to XJ-160 caused bigger plaques on BHK-21 cells and exhibited less neurovirulent in suckling mice than parental virus did. As shown in Figure 3, similar to XJ-160 virus is that all the mutants showed fatal neurovirulence in suckling mice. However, XJ-160 virus killed all animals about 7 days after inoculation. In contrast, BR-146 H, BR-149R and BR-HR only killed 50%, 40% and 80% of mice 10 days after inoculation (Figure 3). Consistence with our data is that the increased binding to cellular HS is associated with loss of neurovirulence of Murray Valley encephalitis virus [28]. In contrast, decreased HS binding resulted in loss of neurovirulence of Theilers murine encephalomyelitis virus [29]. The distinct effects of HS-binding on viral neurovirulence suggest that the two parameters are relatively separable, and that other properties of the virus play an important role for viral neurovirulence.

## Competing interests

The authors declare that they have no competing interests.

## Authors' contributions

WZ carried out the molecular genetic studies, participated in the sequence alignment and drafted the manuscript. SF carried out the immunoassays. YH participated in the sequence alignment. JL participated in the design of the study and performed the statistical analysis. GL conceived of the study, and participated in its design and coordination. All authors read and approved the final manuscript.

## Supplementary Material

Additional file 1 **Figure 1 Infectivity of the mutant viruses in BHK-21 cells**. Cytopathic effect (CPE) 36 h postinfection (p.i.) (*upper panels*), IFA 48 h p.i. (*middle panels*), and plaque-forming assay 24 h p.i. (*lower panels*) of parental virus and the mutants were observed in BHK-21 cells. Click here for file

Additional file 2 **Figure 2 Growth curves of mutant viruses and the parental viruses**. Monolayer of BHK-21 cells at 80% confluency was infected with parental viruses and recombinant viruses at a multiplicity of infection of 0.01. The medium (1 ml) was removed on hours 20, 24, 28, 32, 36 and 44 h p. i., and frozen for later determination of virus titers, and equal volume of fresh medium was added. The virus titers are shown as the mean ± SD of 3 replicate experiments. Click here for file
