# Supplementary figures and images for: Amino acid substitutions in the E2 glycoprotein of Sindbis-like virus XJ-160 confer the ability to undergo heparan sulfate-dependent infection of mouse embryonic fibroblasts
Source: Virol J. 2010 Sep 14;7:225. doi: 10.1186/1743-422X-7-225 (PMC2944170; doi:10.1186/1743-422X-7-225)

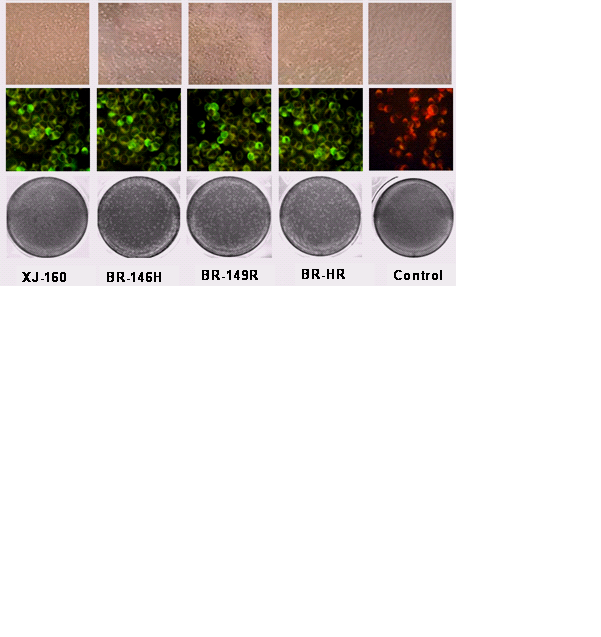

Supplement: Additional file 1 — Figure 1 Infectivity of the mutant viruses in BHK-21 cells. Cytopathic effect (CPE) 36 h postinfection (p.i.) (upper panels), IFA 48 h p.i. (middle panels), and plaque-forming assay 24 h p.i. (lower panels) of parental virus and the mutants were observed in BHK-21 cells. [file 1743-422X-7-225-S1.TIFF]

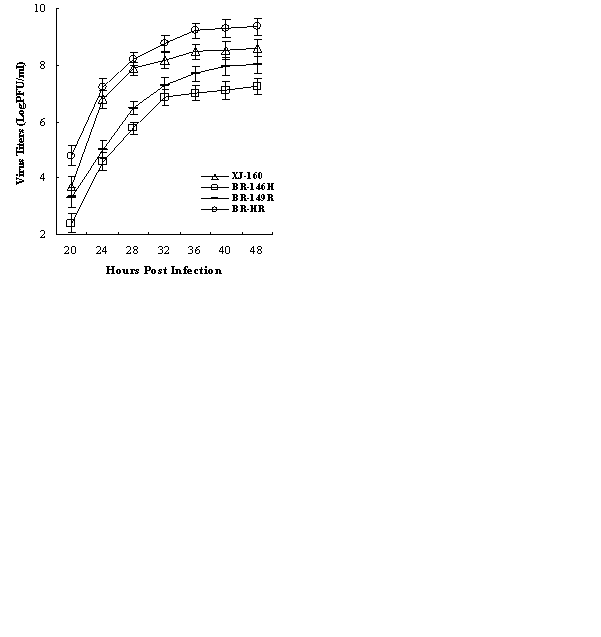

Supplement: Additional file 2 — Figure 2 Growth curves of mutant viruses and the parental viruses. Monolayer of BHK-21 cells at 80% confluency was infected with parental viruses and recombinant viruses at a multiplicity of infection of 0.01. The medium (1 ml) was removed on hours 20, 24, 28, 32, 36 and 44 h p. i., and frozen for later determination of virus titers, and equal volume of fresh medium was added. The virus titers are shown as the mean ± SD of 3 replicate experiments. [file 1743-422X-7-225-S2.TIFF]
